# Choosing the Right Extraction Pattern: First Premolars vs. Second Premolars

**DOI:** 10.7759/cureus.88171

**Published:** 2025-07-17

**Authors:** Ahmed S Khalil, Rawan S Alrehaili, Salwa Hakami, Maram Darraj, Ghada Alshehri, Joud Alrajhi, Basmah Farooqi, Fatimah Alshayook, Yara Abdulbari, Zayan Alsaif, Alanoud Suhaqi

**Affiliations:** 1 Orthodontics, Private Practice, Medina, SAU; 2 Dentistry, Private Practice, Medina, SAU; 3 College of Dentistry, Jazan University, Jazan, SAU; 4 College of Dentistry, King Saud Bin Abdulaziz University for Health Sciences, Riyadh, SAU; 5 College of Dentistry, King Saud bin Abdulaziz University for Health Sciences, Riyadh, SAU; 6 Faculty of Dentistry, King Abdulaziz University, Jeddah, SAU; 7 College of Dentistry, Riyadh Elm University, Riyadh, SAU; 8 Orthodontics and Dentofacial Orthopedics, Ministry of Health, Riyadh, SAU

**Keywords:** airway, anchorage, extraction, first premolar extraction, orthodontic treatment, second premolar extraction, vertical dimension

## Abstract

Premolar extraction is a widely used orthodontic intervention to manage crowding, correct malocclusion, and achieve balanced facial aesthetics. Several factors, including the severity of crowding, facial profile requirements, and anchorage demands, influence the choice between extracting first or second premolars. However, this decision is complicated by the distinct impacts each extraction pattern has on tooth movement, soft tissue profile, vertical dimension, and third molar eruption. The debate continues over which extraction pattern provides more favorable outcomes, highlighting the need for a thorough understanding of their respective advantages and limitations. This review aims to comprehensively analyze the effects of first versus second premolar extractions on anchorage loss, tooth movement, vertical dimensions, lip position, and third molar eruption in order to provide evidence-based insights for optimizing orthodontic treatment outcomes and guiding future research. The findings demonstrate that both extraction patterns have distinct clinical implications and should be selected based on individual patient characteristics, including crowding severity, facial esthetics, anchorage needs, and third molar considerations. Clear aligner therapy requires particular attention to anchorage control, especially following second premolar extraction. Further longitudinal studies are recommended to strengthen clinical guidelines and improve treatment predictability.

## Introduction and background

Premolar extractions have long been a cornerstone of orthodontic treatment, employed to alleviate crowding, correct malocclusions, and enhance facial esthetics while achieving functional occlusion [1,2]. The decision of the extraction pattern is often impacted by the severity of dental crowding, the need for anterior retraction, and the desired soft tissue profile [3]. However, the choice between these two extraction patterns remains a subject of debate among orthodontists due to their differing impacts on dental alignment, facial esthetics, and overall treatment outcomes.

First premolar extractions are typically selected when significant anterior retraction is required, such as in cases of bimaxillary protrusion, protrusive lips, or severe crowding. This pattern provides more space for incisor retraction, thereby influencing lip position and soft tissue profile. On the other hand, second premolar extractions are often chosen when less anterior retraction is needed or a fuller facial profile is preferred. However, this choice can lead to greater mesial movement of the molars, necessitating more rigorous anchorage control. This is particularly relevant in cases of reciprocal anchorage, where the anterior segment is retracted while the posterior segment moves mesially. In such cases, the extraction of second premolars tends to yield more pronounced mesial molar movement compared to first premolar extraction because the ratio of the buccal segment to front teeth is smaller, thereby affecting the anchorage balance. Building on this, it was hypothesized that posterior anchorage requirements differ between the maxillary and mandibular arches due to variations in their anatomical structures [4]. These differences in anchorage requirements highlight the need for careful extraction pattern selection based on individual patient characteristics and treatment objectives [5,6]. Additionally, the type of appliance used in orthodontic treatment plays a crucial role in determining the outcomes of anchorage in extraction cases [7,8]. With the increasing popularity of clear aligner therapy, questions arise about how different extraction patterns influence anchorage loss, tooth movement efficiency, and overall treatment stability when compared to a conventional fixed appliance. Beyond tooth movement and anchorage requirements, premolar extractions also influence facial vertical dimensions, lip position, and the eruption patterns of third molars. For instance, changes in facial vertical dimension are closely related to the extraction pattern, especially in patients with hyperdivergent facial types [9]. Similarly, the choice of extraction pattern affects lip position and soft tissue profile, which are critical for achieving esthetic harmony [10]. Furthermore, the extraction of premolars alters the available space for third molar eruption, potentially affecting the impaction risk and eruption angulation [11,12]. These multifaceted consequences underscore the need for comprehensive treatment planning to optimize both functional and esthetic outcomes.

Although numerous studies have examined the effects of first versus second premolar extractions, the evidence remains inconclusive and contradictory. Variations in study designs, sample sizes, measurement techniques, and follow-up periods contribute to inconsistent findings. This variability complicates clinical decision-making and highlights the need for a review of the literature. As a result, a comprehensive appraisal of existing studies is warranted to better understand and reconcile the divergent findings. This narrative review aimed to comprehensively evaluate the current evidence on how first and second premolar extractions impact orthodontic outcomes, including anchorage loss, tooth movement, vertical dimensions, lip changes, third molar eruption patterns, and potential changes in airway dimensions.

## Review

Search strategy

A search of electronic databases, including PubMed, Scopus, and Web of Science, was conducted to identify studies relevant to the effects of first versus second premolar extractions. The search included articles published up to April 2025, using combinations of keywords and MeSH terms such as "first premolar extraction," "second premolar extraction," "orthodontic treatment," "anchorage loss," "facial vertical dimension," "lip profile," "third molar eruption," "airway," and "clear aligners." Search terms included combinations like ("first premolar extraction" OR "second premolar extraction") AND ("orthodontics" OR "orthodontic treatment") AND ("anchorage loss" OR "dental movement" OR "facial esthetics" OR "lip position" OR "third molar eruption" OR "clear aligners"). A broad range of study designs was considered, including randomized controlled trials, cohort studies, retrospective studies, and finite element analyses, to capture comprehensive evidence on the topic. The findings from the included studies were narratively synthesized to provide an integrated overview of the evidence regarding the clinical implications of first versus second premolar extractions.

Comparative effects on anchorage and arch dimensions

The choice between first and second premolar extractions plays a crucial role in influencing anchorage loss, molar movements, and overall arch dimensional changes during orthodontic treatment. Understanding the differential effects of these extraction patterns helps optimize space closure mechanics and achieve desired occlusal outcomes. Haque et al. conducted a retrospective study to compare the amount of anchorage loss following the extraction of maxillary first premolars versus maxillary second premolars [13]. The study involved 60 adolescent patients with Class I, mild Class II, or mild Class III malocclusions and mild-to-moderate crowding, treated with a lack of anchorage preparation. Anchorage loss was evaluated by measuring the anchorage loss of the maxillary first permanent molar. The results indicated that the mean anchorage loss of the maxillary first molar was 4.7 mm in the first group and 4.6 mm in the second group, with no statistically significant difference between the two groups. The study concluded that there was no difference in anchorage loss between extracting maxillary first premolars and maxillary second premolars in adolescent patients. In contrast, George et al. explored the effects of extraction patterns on incisor and molar movements specifically in growing patients with Class II Division 1 malocclusion [14]. This study examined 54 patients aged 10-17, treated using Tweed directional force mechanics, J-hook headgears, and Class II elastics or Saif springs. Patients were divided into two groups: extraction of maxillary and mandibular first premolars (4/4) or extraction of maxillary first and mandibular second premolars (4/5), with 27 patients in each group. The findings revealed that extracting mandibular second premolars resulted in greater mesial movement of the mandibular first molars and less distal movement of the mandibular incisors compared to first premolar extractions. Specifically, second premolar extractions led to 1.6 mm less incisor retraction and 0.7 mm more molar protraction. Interestingly, no significant differences were observed between the two groups in vertical eruption of molars or changes in the mandibular plane angle, indicating no wedge effect. The study concluded that mandibular second premolar extractions are more effective for Class II molar correction due to enhanced mesial molar movement and reduced incisor retraction, without affecting the vertical dimension. Further contributing to the understanding of extraction patterns, Shearn and Woods investigated the cephalometric and arch dimensional changes in the mandibular arch associated with the extraction of lower first versus lower second premolars when using a fixed orthodontic appliance [15]. This study analyzed the pretreatment and post-treatment records of 73 patients treated by an experienced orthodontist. Eighteen patients underwent extraction of lower first premolars, while 55 underwent extraction of lower second premolars. Among the lower second premolar group, 29 had upper first premolars extracted, and 26 had upper second premolars extracted. In contrast, all patients in the lower first premolar group also had upper first premolars removed. The study identified several pretreatment factors influencing the choice of extraction pattern, including incisal overjet, molar relationship, and vertical facial pattern. It was observed that different extraction patterns led to varied arch dimensional changes. Notably, the extraction of lower second premolars resulted in a greater reduction of intermolar arch width compared to lower first premolars. Contrary to common assumptions, premolar extraction did not consistently cause a retrusive effect on the incisors. In fact, incisor proclination was observed across all extraction groups. The study also noted substantial individual variation in incisor and molar movements, regardless of the extraction pattern.

Impact on tooth-size discrepancy

Tooth-size discrepancy refers to the mismatch in the mesiodistal dimensions of the maxillary and mandibular teeth, which can affect the alignment, occlusion, and overall esthetics of the dental arches [16]. It is typically assessed using Bolton's analysis, which compares the total width of teeth in the lower arch to the upper arch. An imbalance in these ratios can lead to spacing, crowding, or improper intercuspation, influencing the stability and function of the occlusion [17,18]. Understanding tooth-size discrepancy is particularly crucial when planning orthodontic treatment involving premolar extractions, as different extraction patterns can significantly impact the resulting tooth-size ratios [19]. Selecting the appropriate extraction pattern helps achieve an optimal arch coordination and occlusal relationship, minimizing the need for compensatory mechanics or post-treatment adjustments. Saatci and Yukay evaluated whether extracting four premolars in orthodontic treatment affects tooth-size discrepancies and if different extraction patterns influence the severity of these discrepancies [20]. The study analyzed pretreatment dental casts of 50 patients with balanced mandibular and maxillary arch dimensions, using Bolton's analysis before and after hypothetical extractions. The extraction combinations included all first premolars, all second premolars, upper first and lower second premolars, and upper second and lower first premolars. Results showed that extracting all first premolars significantly increased tooth-size discrepancies, while other combinations did not. The first premolar extraction produced the most severe discrepancies, whereas extracting all second premolars resulted in the least. The study concluded that second premolar extractions minimize tooth-size discrepancies, offering a strategic consideration for orthodontic treatment planning. Expanding on these findings, Kumar et al. investigated the impact of premolar extractions on Bolton overall ratios and tooth-size discrepancies in a North Indian population [21]. The study analyzed pretreatment dental casts of 120 orthodontic patients (60 males and 60 females) using Bolton's analysis. Hypothetical extractions were simulated for four patterns: all first premolars, all second premolars, upper first and lower second premolars, and upper second and lower first premolars. The findings revealed a mild maxillary tooth material excess in the population. Extracting premolars in any pattern increased this maxillary excess, with no significant differences between genders. The results highlighted that maxillary tooth mass might increase after extraction, influencing treatment planning.

Vertical dimension outcomes

Vertical dimension refers to the vertical height of the face, primarily influenced by the position of the maxilla and mandible, as well as the inclination of the occlusal and mandibular planes. In orthodontics, maintaining or modifying the facial vertical dimension is crucial for achieving balanced facial esthetics and functional occlusion [22,23]. Different premolar extraction patterns have been hypothesized to impact the vertical dimension by altering the position of incisors and molars, thus affecting the mandibular plane angle and facial height. Understanding these effects is vital for selecting the appropriate extraction pattern, especially in patients with hyperdivergent or hypodivergent facial types. Burashed compared changes in facial vertical dimension and incisor positioning after orthodontic treatment with first premolar extractions (Group A), second premolar extractions (Group B), and non-extraction (Group C). Vertical changes were assessed using mandibular plane angle and the position of the incisors on pre-and post-treatment lateral cephalograms. Results showed no significant differences in mandibular plane angle changes across all groups, regardless of extraction pattern or non-extraction. However, upper and lower incisors were significantly retracted in Groups A and B but protracted in Group C. Additionally, upper incisors retroclined more in Group A compared to Group B, while Group C showed significant proclination. The study concluded that different premolar extraction patterns do not impact vertical dimension changes [24]. Extraction decisions should be based on desired incisor movements rather than controlling the vertical dimension. Similarly, Wang et al. compared vertical changes in Class I malocclusion patients treated with two extraction patterns: maxillary first and mandibular second premolars (4/5, Group A) versus all first premolars (4/4, Group B). The study retrospectively analyzed cephalometric records of 47 patients in Group A and 46 in Group B. Eight skeletal and 10 dental cephalometric measurements were used to evaluate vertical changes. Results showed that both groups experienced significant vertical changes after orthodontic treatment, but no significant differences were observed between the two extraction patterns. The expected wedge effects from the mesial movement of posterior teeth were offset by posterior teeth extrusion and residual growth potential. The study concluded that neither extraction pattern significantly affected vertical dimensions, suggesting flexibility in extraction choices for Class I malocclusion without impacting facial vertical proportions [25]. Supporting these findings, Kim et al. evaluated the impact of first versus second premolar extractions on facial vertical dimension in patients with Class I malocclusion and hyperdivergent facial types. The second premolar extraction group showed more mesial movement of the maxillary and mandibular first molars and less incisor retraction compared to the first premolar extraction group. Although both groups experienced increased anterior facial height, there were no significant differences in overall facial vertical dimension changes between the groups, except for the maxillomandibular plane angle and SN to palatal plane angle. The study concluded that neither first nor second premolar extractions led to a decrease in facial vertical dimension, refuting the hypothesis that second premolar extractions reduce facial height by minimizing the wedge effect [26].

Influence on profile and lip position

The choice between first and second premolar extractions can significantly influence soft tissue profiles and lips, impacting overall facial esthetics. The soft tissue profile is primarily determined by the position of the lips, nose, and chin relative to the underlying dental and skeletal structures. Changes in lip curvature, including the nasolabial angle and lip prominence, directly affect facial harmony and the perception of attractiveness [27]. The retraction of incisors following premolar extractions can alter the position and curvature of the lips [28,29]. These changes are influenced by factors such as the initial thickness of the lips, the amount of crowding, and the mechanics of space closure. Wholley and Woods investigated the impact of different premolar extraction patterns on upper and lower lip curvature following orthodontic treatment. The study aimed to determine if specific extraction sequences and the removal of all first premolars, all second premolars, or upper first and lower second premolars produced consistent changes in facial profile [30]. The findings revealed that changes in lip curvature were not solely determined by the choice of extraction pattern. Instead, there was considerable individual variation in the degree of change. The study concluded that the soft tissue response depended on the patient's initial lip morphology, the interplay between dental and skeletal changes, and the orthodontist's skill in managing extraction spaces. The authors suggested that clinicians could effectively manage either first or second premolar extraction spaces without compromising the facial profile, emphasizing the importance of individualized treatment planning. Building on these findings, Wholley and Woods evaluated the effects of orthodontic treatment using three different premolar extraction patterns: extraction of all first premolars, extraction of upper first and lower second premolars, and extraction of all second premolars. They compared findings from three previous studies to assess changes in tooth movement and soft tissue profiles. The study revealed significant individual variation in molar movement, incisor retraction, and changes in the curvature of the upper and lower lips across all extraction patterns. The degree of crowding before treatment and the space remaining after initial alignment were identified as key factors influencing tooth position changes. Additionally, changes in lip curvature were affected by the initial thickness of the lips at the vermilion border. The authors concluded that factors beyond the choice of extraction pattern, such as pretreatment crowding and lip thickness, play a significant role in influencing molar, incisor, and lip positions during orthodontic treatment [31]. Saelens and De Smit compared orthodontic outcomes among three groups: extraction of first premolars, extraction of second premolars, and non-extraction using Begg appliances. The study assessed initial crowding, movement of incisors and molars, soft tissue profile changes, and clinical outcomes using the Peer Assessment Rating Index. The group with first premolar extractions showed the highest pretreatment crowding, leading to a higher Peer Assessment Rating score compared to the other groups. The group with second premolar extractions exhibited greater incisor protrusion and smaller inter-incisal angles. During treatment, upper incisors moved backward in both extraction groups, while the non-extraction group showed forward movement of incisors and lips. Molar movement was highest in the second premolar extraction group. All groups maintained favorable facial profiles and achieved optimal clinical outcomes with low post-treatment Peer Assessment Rating scores [32]. Similarly, Omar et al. conducted a retrospective clinical study to compare changes in the soft tissue profile following orthodontic treatment with the extraction of either first or second premolars. The study included two groups: one group underwent extraction of all first premolars, while the other had all second premolars removed. The researchers evaluated changes in the nasolabial angle and the position of the upper and lower lips relative to the E-plane before and after treatment. They also considered factors such as age, gender, upper lip thickness, facial convexity, facial axis, arch crowding, molar anchorage usage, and the extent of maxillary and mandibular incisor retrusion during treatment. The results showed that when accounting for facial convexity, facial axis, use of maxillary anchorage, and incisor retrusion, there was no significant difference in the change of the nasolabial angle between the two groups, with a minimal difference of 0.67 degrees. Additionally, the changes in the upper and lower lip positions relative to the E-plane were similar between the groups. The study concluded that the choice of extracting either first or second premolars did not result in significant differences in soft tissue profile changes [33].

Impact on the airway

The influence of premolar extraction on pharyngeal airway dimensions has gained increasing attention due to its potential implications for respiratory function and overall treatment planning. Orthodontic interventions involving tooth extractions, particularly when accompanied by significant anterior retraction, may affect tongue posture and surrounding soft tissues, which in turn can alter airway morphology [34]. While numerous studies have evaluated pharyngeal airway changes following premolar extractions in general, very limited evidence exists directly comparing the effects of first versus second premolar extractions on airway dimensions. A recent study by Badepalli et al. examined airway changes following the extraction of either four or five premolars, demonstrating significant reductions in airway width and volume in both groups. Yet, no significant differences were observed between the two extraction patterns [35]. Similarly, Mladenovic et al. evaluated changes in the oral cavity proper using CBCT and reported that patients undergoing second premolar extractions showed less volumetric expansion of the oral cavity compared to non-extraction patients, potentially impacting tongue posture and airway space [36]. However, it is noteworthy that most available studies do not distinguish between first and second premolar extractions when evaluating airway-related outcomes. For example, Joy et al. assessed airway changes in adult patients undergoing orthodontic treatment with and without extractions but concluded that no consistent airway reduction could be directly attributed to extractions alone, regardless of pattern [37]. Moreover, Cho et al. found that pharyngeal narrowing was more likely in hyperdivergent individuals following premolar extractions, particularly in the glossopharyngeal region [38]. Nagmode et al. reported a reduction in inferior airway space following first premolar extractions in bimaxillary protrusion patients, further indicating that treatment-induced changes in tongue position and alveolar structure may influence pharyngeal space [39]. Yet, such studies remain insufficient for establishing definitive differences between first and second premolar extraction patterns with regard to airway impact [40,41]. Existing evidence suggests that no strong comparative data currently exist to determine whether first or second premolar extractions are more detrimental or favorable in this regard.

Consequences on third molar angulation and space available

Third molars are the most frequently impacted teeth due to space limitations in the dental arch. Their eruption pattern, angulation, and position can be significantly influenced by the available space in the posterior dental arch, which is affected by premolar extractions performed during orthodontic treatment. Extraction of first or second premolars may alter the mesial movement of molars and the distribution of space in the dental arch, potentially impacting third molar eruption space, angulation, and risk of impaction. Miclotte et al. investigated the impact of the extraction pattern on eruption space, position, and angulation of third molars during orthodontic treatment. Results showed that premolar extractions significantly increased the eruption space and improved the vertical position of third molars compared to non-extraction cases. However, changes in inclination, relationship with the inferior alveolar canal, and mineralization degree did not significantly differ between the extraction and non-extraction groups. The study concluded that premolar extractions positively influence the available space for eruption and vertical position of third molars but do not impact their angulation [42]. Similarly, Tarazona et al. compared changes in mandibular third molar angulation and position among patients treated with first premolar extractions, second premolar extractions, or non-extraction orthodontic treatments. Results showed that third molar angulation improved over time in all groups, with greater disinclusion observed in extraction cases. There were no significant differences in third molar inclination or impaction risk when comparing first and second premolar extraction groups. The gonial angle decreased with age across all groups. The study concluded that while premolar extractions facilitate third molar disinclusion, they do not significantly influence angulation or impaction risk [43]. Other factors likely influence third molar position, making it difficult to establish a predictive impaction model.

Clear aligners: does the extraction pattern matter?

Clear aligner therapy has gained popularity in orthodontics due to its esthetic appeal and improved patient comfort. However, anchorage control and dental movements can be more challenging with clear aligners compared to traditional fixed appliances, especially in cases requiring premolar extractions [44-46]. The choice between first and second premolar extractions significantly impacts anchorage loss, molar tipping, and overall dental alignment [47,48]. Qiang et al. investigated anchorage loss in posterior teeth during clear aligner treatment with first versus second premolar extractions in the maxillary and mandibular arches. Using finite element models, the study simulated anterior en-masse retraction for two extraction patterns: first premolar extractions (Model 1) and second premolar extractions (Model 2). Results indicated that first premolar extractions resulted in greater lingual inclination of anterior teeth and less mesial tipping of posterior teeth compared to second premolar extractions. The closer the teeth were to the extraction space, the more pronounced the tipping. Maxillary posterior teeth displayed a greater mesial tipping tendency than mandibular teeth. Additionally, periodontal ligament stress was more uniformly distributed in first premolar extractions. The study concluded that anchorage preparation is crucial to reduce the roller coaster effect, particularly in second premolar extractions, with a recommendation for stronger anchorage support in maxillary posterior teeth [49]. Similarly, Tang et al. compared relative anchorage loss in mandibular first versus second premolar extraction cases treated with clear aligner therapy in patients with bimaxillary protrusion and mild crowding. Relative anchorage loss was evaluated as the percentage of drift relative to the total movement of the molar and canine. Results showed that first molars moved mesially by 2.01 mm with 25% relative anchorage loss in the first premolar extraction group, whereas they moved 3.25 mm with 40% relative anchorage loss in the second premolar extraction group. The second premolar extraction group experienced significantly more anchorage loss. The study concluded that second premolar extractions resulted in greater anchorage loss compared to first premolar extractions [50]. The findings suggest using a relative anchorage loss-based treatment planning approach for mandibular premolar extractions in clear aligner therapy (Table 1).

**Table 1:** Summary of the differences between first and second premolar extractions

Outcome Category	First Premolar Extraction	Second Premolar Extraction
Anchorage Loss	Less anchorage loss; more controlled mesial movement of molars	Greater anchorage loss due to increased mesial movement of molars, requiring enhanced anchorage strategies
Tooth Movement	Greater incisor retraction, leading to more pronounced anterior retraction and lip retrusion	Less incisor retraction
Facial Vertical Dimensions	Less impact on vertical dimensions	Slightly more impact on vertical dimensions
Lip Changes	More significant retraction of lips, influencing a more concave soft tissue profile.	Less lip retraction, maintaining a fuller soft tissue profile.
Airway	Very limited evidence exists directly comparing the effects of first versus second premolar extractions on airway	
Third Molar Eruption Patterns	Less space for third molar eruption	More space for third molar eruption

Clinical implications and future research

The results emphasize the importance of individualized treatment planning when selecting premolar extraction patterns, particularly in terms of achieving esthetic soft tissue profiles and optimizing occlusal outcomes. Orthodontists should consider patient-specific factors such as initial crowding severity, lip thickness, facial growth patterns, and treatment objectives when determining the extraction sequence. The findings also suggest the need for enhanced anchorage strategies in clear aligner therapy, especially when second premolar extractions are planned. From a policy perspective, the variability in outcomes underscores the necessity for standardized protocols and guidelines in orthodontic treatment planning, particularly concerning extraction decisions. Establishing evidence-based guidelines would aid clinicians in making informed choices and ensuring consistent patient outcomes. Additionally, the implications of third molar eruption space following premolar extractions could influence recommendations for third molar management in orthodontic patients.

**Figure 1:**
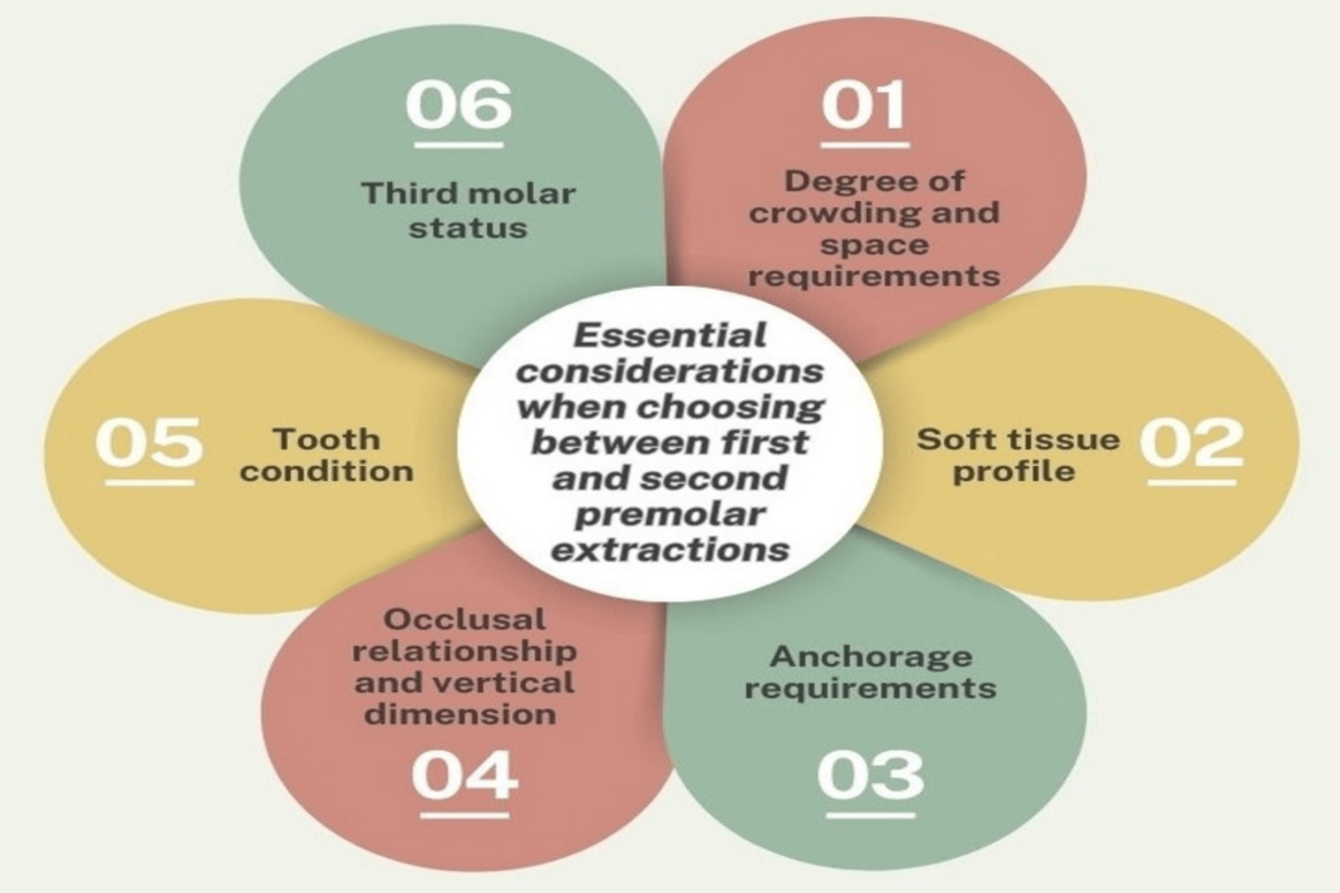
The key factors to consider when deciding between first or second premolar extractions in orthodontic treatment Created with the help of Canva (www.canva.com)

For future research, there is a need for well-designed prospective longitudinal studies with larger, more diverse samples to validate the current findings and establish more definitive conclusions about the long-term effects of different extraction patterns. Utilizing three-dimensional imaging techniques would enhance the accuracy of soft tissue and dental movement assessments. Standardizing outcome measures and incorporating control for confounding variables, such as growth patterns and lip thickness, would improve the reliability of comparative studies. Moreover, future research should explore the psychological and quality-of-life impacts associated with esthetic changes following premolar extractions. Such in-depth investigations would provide a more refined understanding of the effects of premolar extraction patterns, helping inform evidence-based orthodontic practice (Figure 1).

Limitations

The evidence included in this review presents several limitations that may influence the validity and generalizability of the findings. One significant limitation is the retrospective nature of many studies, which inherently carries a risk of selection bias and limits the ability to establish causal relationships between premolar extraction patterns and their effects on dental and soft tissue outcomes. Additionally, the heterogeneity in study designs, including differences in sample sizes, age groups, treatment durations, and types of orthodontic appliances used, complicates direct comparison and synthesis of results. Furthermore, a majority of the studies relied on two-dimensional cephalometric analysis, which may not accurately capture the three-dimensional changes in dental and soft tissue profiles. The absence of long-term follow-up data also restricts the evaluation of post-treatment stability and the potential relapse of orthodontic outcomes. Another limitation is the underrepresentation of certain populations, as most studies were conducted within specific ethnic or demographic groups, thereby limiting the applicability of the findings to broader populations. Finally, the lack of standardized outcome measures and inconsistent reporting of confounding variables, such as initial crowding severity, lip thickness, and facial growth patterns, could lead to biased interpretations of the effects of different extraction patterns. This review is also subject to certain limitations related to the review process itself. Although efforts were made to include a comprehensive range of studies, there is a possibility of publication bias, where positive or significant findings are more likely to be published than non-significant results. Additionally, variations in study quality and methodological rigor were observed across the included studies, and although no formal quality assessment tool was employed, this could influence the strength of the conclusions drawn. The exclusion of non-English studies may also have limited the diversity of the evidence base. Furthermore, the narrative synthesis approach used to integrate findings may introduce subjectivity in interpreting the results, as opposed to a quantitative meta-analysis, which could provide a more robust summary of the effects.

## Conclusions

The findings of this review emphasize the complexity and individuality of orthodontic treatment responses, revealing that no single extraction pattern delivers superior outcomes. Instead, the choice between first and second premolar extractions should be guided by individualized treatment objectives, considering factors such as initial crowding, lip thickness, facial growth pattern, and desired esthetic outcomes. The review highlights that first premolar extractions tend to produce more significant retraction of incisors, influencing lip position and facial profile, whereas second premolar extractions are associated with increased mesial molar movement and greater anchorage demands, particularly in clear aligner therapy. The current evidence is insufficient to establish whether first or second premolar extractions have a more favorable or detrimental impact on the pharyngeal airway. Additionally, the evidence suggested that premolar extractions positively influence the third molar eruption space without significantly affecting angulation or impaction risk. However, the review also poses several limitations within the existing literature, including methodological variability, limited long-term follow-up, and a lack of standardized outcome measures, which restrict the generalizability of the findings.
